# Supplementary material for: Compositing effects for high thermoelectric performance of Cu2Se-based materials
Source: Nat Commun. 2023 Apr 27;14:2410. doi: 10.1038/s41467-023-38054-y (PMC10140174; doi:10.1038/s41467-023-38054-y)
Supplement: Supplementary file 1 — Supplementary Information [file 41467_2023_38054_MOESM1_ESM.pdf]

Supplementary Information for

Compositing Effects for High Thermoelectric Performance of Cu_2Se-Based Materials

Zhifang Zhou[1], Yi Huang[2,3], Bin Wei[1,4], Yueyang Yang[1], Dehong Yu[5], Yunpeng Zheng[1], Dongsheng He[6], Wenyu Zhang[1], Mingchu Zou[1], Jin-Le Lan[7,*], Jiaqing He[2], Ce-Wen Nan[1], Yuan-Hua Lin[1,*]

1. State Key Laboratory of New Ceramics and Fine Processing, School of Materials Science and Engineering, Tsinghua University, Beijing 100084, China
2. Shenzhen Key Laboratory of Thermoelectric Materials, Department of Physics, Southern University of Science and Technology, Shenzhen 518055, China
3. Analytical Instrumentation Center, Hunan University, Changsha 410000, China
4. Henan Key Laboratory of Materials on Deep-Earth Engineering, School of Materials Science and Engineering, Henan Polytechnic University, Jiaozuo 454000, China
5. Australian Nuclear Science and Technology Organisation, Lucas Heights, New South Wales 2234, Australia
6. Core Research Facilities, Southern University of Science and Technology, Shenzhen 518055, China
7. State Key Laboratory of Organic-Inorganic Composites, College of Materials Science and Engineering, Beijing University of Chemical Technology, Beijing 100029, China

Corresponding authors:

E-mail addresses: lanjl@mail.buct.edu.cn (J.-L. Lan), linyh@mail.tsinghua.edu.cn (Y.-H. Lin)

This file includes:

Supplementary Text

Supplementary Figures 1-12

Supplementary Tables 1-3

Supplementary References

Supplementary Text

Calculation for weighted mobility

Weighted mobility (electron mobility weighted by the density of electronic states, μ_w) can describe the inherent electronic transport properties. A simple calculation method from measured Seebeck coefficient (S) and electrical conductivity (σ) was proposed by Snyder et al[1], and the equation is expressed as,

$$\mu_w = \frac{3h^3\sigma}{8\pi e(2m_e k_B T)^{3/2}}\left[\frac{\exp\left(\frac{|S|}{k_B/e}-2\right)}{1+\exp\left[-5\left(\frac{|S|}{k_B/e}-1\right)\right]}+\frac{\frac{3}{\pi^2}\frac{|S|}{k_B/e}}{1+\exp\left[5\left(\frac{|S|}{k_B/e}-1\right)\right]}\right]$$

where h, k_B, m_e stand for Planck constant, Boltzmann constant, and electron mass, respectively. The weighted mobility of $0.9Cu_2Se$-0.1BPCCSO-x wt% graphene composites (x=0, 0.01, 0.02, 0.03, 0.035, 0.04) in this work was calculated by the above equation.

Calculation for Lorentz number

The calculations of Lorentz number (L) are based on the assumption of the single band (SPB) mode and acoustic phonon scattering with the scattering factor, r=–1/2. The correlations are expressed as follows[2],

$$S = \mp\frac{k_B}{e}\left[\frac{(r+5/2)F_{r+3/2}(\eta)}{(r+3/2)F_{r+1/2}(\eta)}-\eta\right]$$

$$L = \left(\frac{k_B}{e}\right)^2\left\{\frac{(r+7/2)F_{r+5/2}(\eta)}{(r+3/2)F_{r+1/2}(\eta)}-\left[\frac{(r+5/2)F_{r+3/2}(\eta)}{(r+3/2)F_{r+1/2}(\eta)}\right]^2\right\}$$

$$F_n(\eta)=\int_0^{\infty}\frac{x^n\mathrm{d}x}{1+\exp(x-\eta)}$$

$$\eta=\frac{E_{\mathrm{F}}}{k_{\mathrm{B}}T}$$

$$x=\frac{E}{k_{\mathrm{B}}T}$$

Calculation for elastic properties and phonon mean free path

Through measuring sound velocity, the elastic properties including Young's modulus (E), Poisson ratio (v_{p}), Grüneisen parameter (γ) are obtained. The longitudinal sound velocity (v_{l}) and shear sound velocity (v_{s}) were measured at room temperature via an ultrasonic pulse-echo method using an Olympus 5072 PR pulser/receiver. The average sound velocity (v_{a}), v_{p}, γ and phonon mean free path (l_{ph}) were calculated as follows[2],

$$\upsilon_{\mathrm{a}}=\left[\frac{1}{3}\left(\frac{1}{v_{\mathrm{l}}^3}+\frac{2}{v_{\mathrm{s}}^3}\right)\right]^{-\frac{1}{3}}$$

$$E=\frac{\rho v_{\mathrm{s}}^2\left(3v_{\mathrm{l}}^2-4v_{\mathrm{s}}^2\right)}{\left(v_{\mathrm{l}}^2-v_{\mathrm{s}}^2\right)}$$

$$\nu_{\mathrm{p}}=\frac{1-2\left(v_{\mathrm{s}}/v_{\mathrm{l}}\right)^2}{2-2\left(v_{\mathrm{s}}/v_{\mathrm{l}}\right)^2}$$

$$\gamma=\frac{3}{2}\left(\frac{1+\nu_{\mathrm{p}}}{2-3\nu_{\mathrm{p}}}\right)$$

$$l_{\mathrm{ph}}=\frac{3\kappa_{\mathrm{l}}}{C_{\mathrm{p}}\rho v_{\mathrm{a}}}$$

Measurement of bending strength

The testing of bending strength was measured by a universal test machine

(Shimadzu, EZ-100, Japan) in a three-point bending format with a constant loading speed of 0.75 mm min^{-1}, and it was calculated by,

$$\sigma_b = \frac{3Fl}{2bd^2}$$

where σ_b, F, l, b, and d stand for bending strength, maximum load value at fracture, the distance between supports (here l=10 nm), width (here b=4 nm), and height (hear d=3 nm), respectively.

Supplementary Figures 1-12

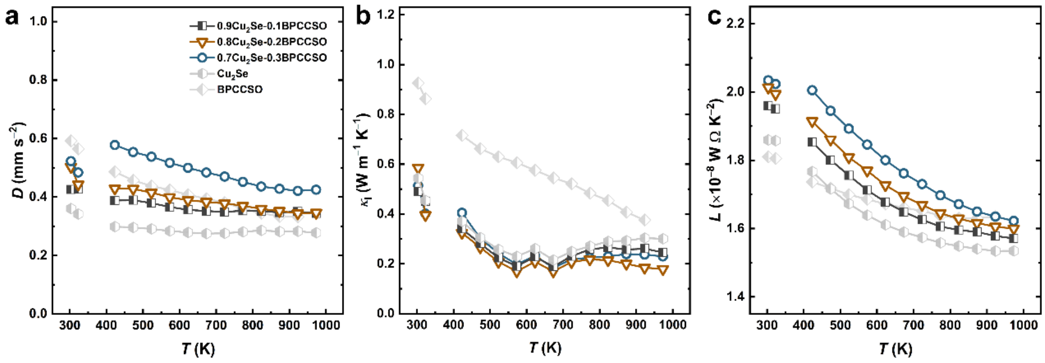

Supplementary Fig. 1 | Temperature-dependent thermal transport properties of Cu_2Se-BPCCSO composites, Cu_2Se and BPCCSO. a, Thermal diffusivity (*D*). **b,** Lattice thermal conductivity (κ_l). **c,** Lorentz number (*L*).

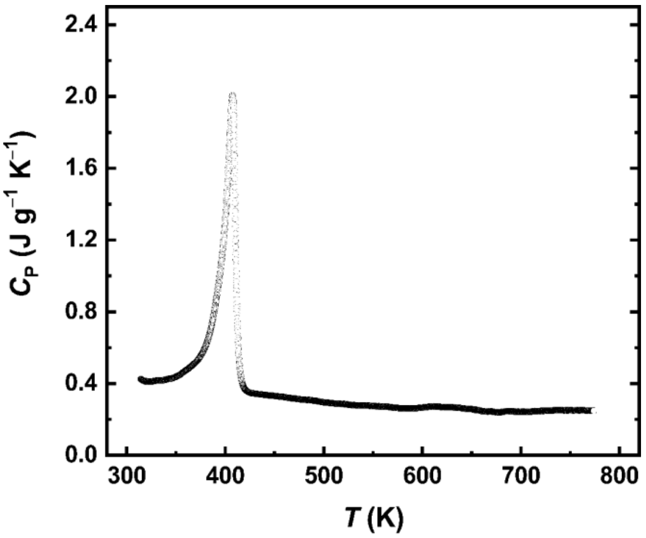

Supplementary Fig. 2 | Temperature-dependent specific heat capacity (*C_p*) for Cu_2Se.

The C_p of BPCCSO was estimated according to the Neumann-Kopp rule, and C_p values of Cu_2Se-BPCCSO composites, $0.9Cu_2Se$-0.1BPCCSO-x wt% graphene composites (x=0.01, 0.02, 0.03, 0.035, 0.04) were also estimated according to the Neumann-Kopp rule based on the measured C_p of Cu_2Se.

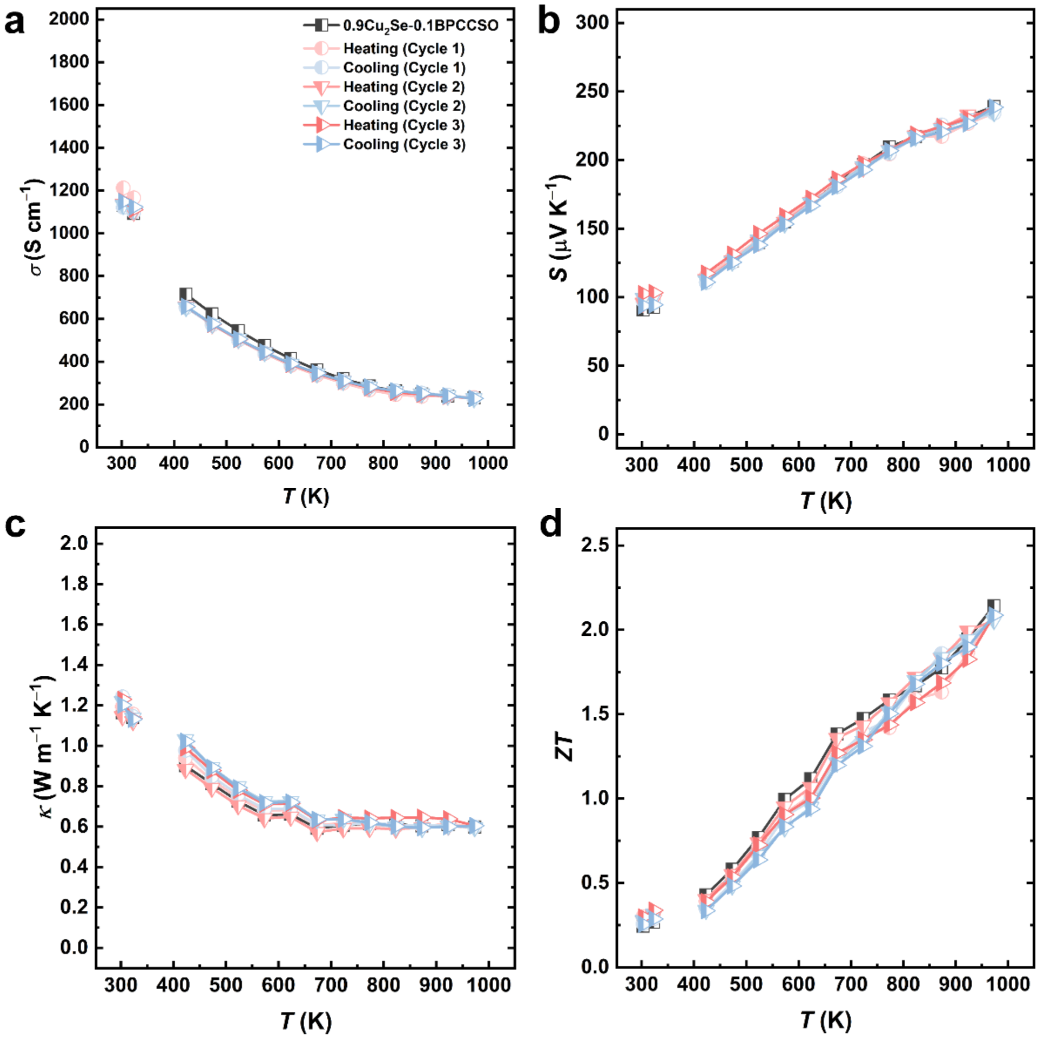

Supplementary Fig. 3 | Temperature-dependent thermoelectric properties of 0.9Cu_2Se-0.1BPCCSO composite under heating and cooling cycles. a, Electrical conductivity (σ). **b,** Seebeck coefficient (S). **c,** Thermal conductivity (κ). **d,** ZT.

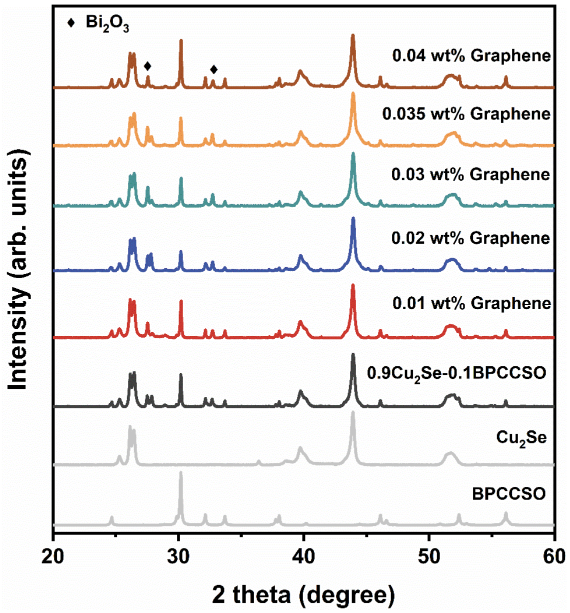

Supplementary Fig. 4 | Phase identification. X-ray diffraction (XRD) patterns of $0.9Cu_2Se$-0.1BPCCSO-x wt% graphene composites (x=0, 0.01, 0.02, 0.03, 0.035, 0.04), BPCCSO and Cu_2Se. The samples were fine powders crushed and ground from the sintered bulks. 2 theta represents the diffraction angle range in the X-ray diffraction test and arb. units means arbitrary units.

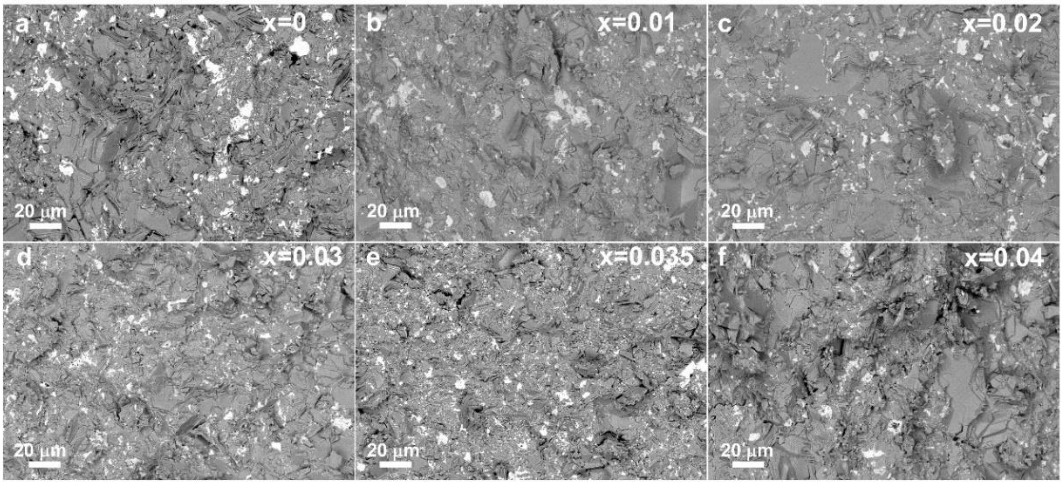

Supplementary Fig. 5 | Microstructure. a-f, Scanning electron microscopy (SEM) images under backscattering electron imaging (BEI) mode showing fracture microstructure of 0.9Cu_2Se-0.1BPCCSO-x wt% graphene composites (x=0, 0.01, 0.02, 0.03, 0.035, 0.04).

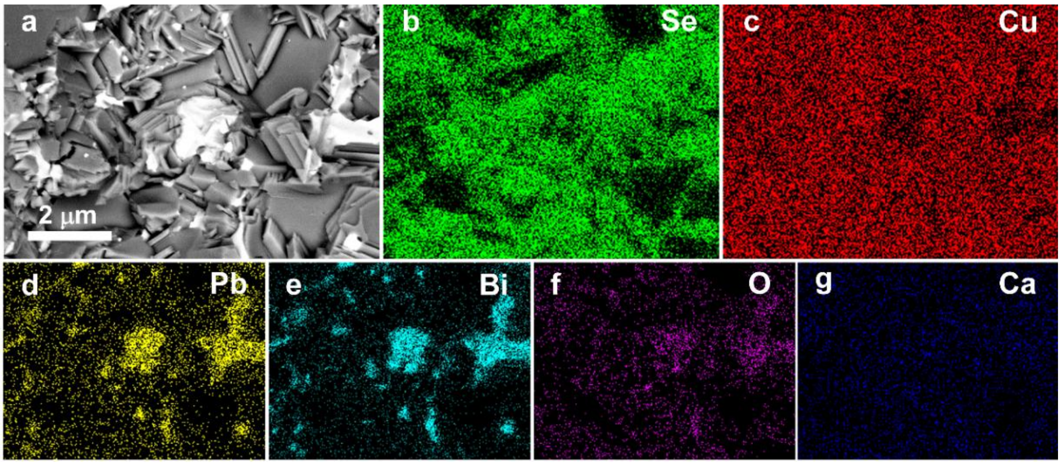

Supplementary Fig. 6 | Microstructure and the corresponding elemental mapping images. a, Scanning electron microscopy (SEM) image of 0.9Cu_2Se-0.1BPCCSO-0.035 wt% graphene composite under backscattering electron imaging (BEI) mode. **b-g,** Energy dispersive spectroscopy (EDS) mapping images of Se, Cu, Pb, Bi, O, Ca.

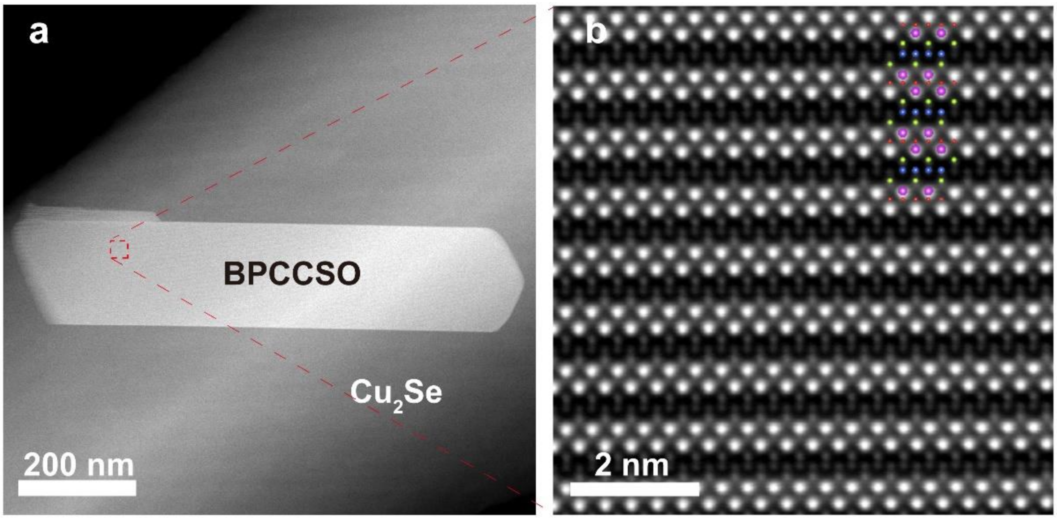

Supplementary Fig. 7 | High-angle annular dark field (HAADF)-Scanning transmission electron microscopy (STEM) images. a, Low-magnification HAADF-STEM image showing area containing BPCCSO and Cu_2Se. **b,** High-resolution HAADF-STEM image of BPCCSO.

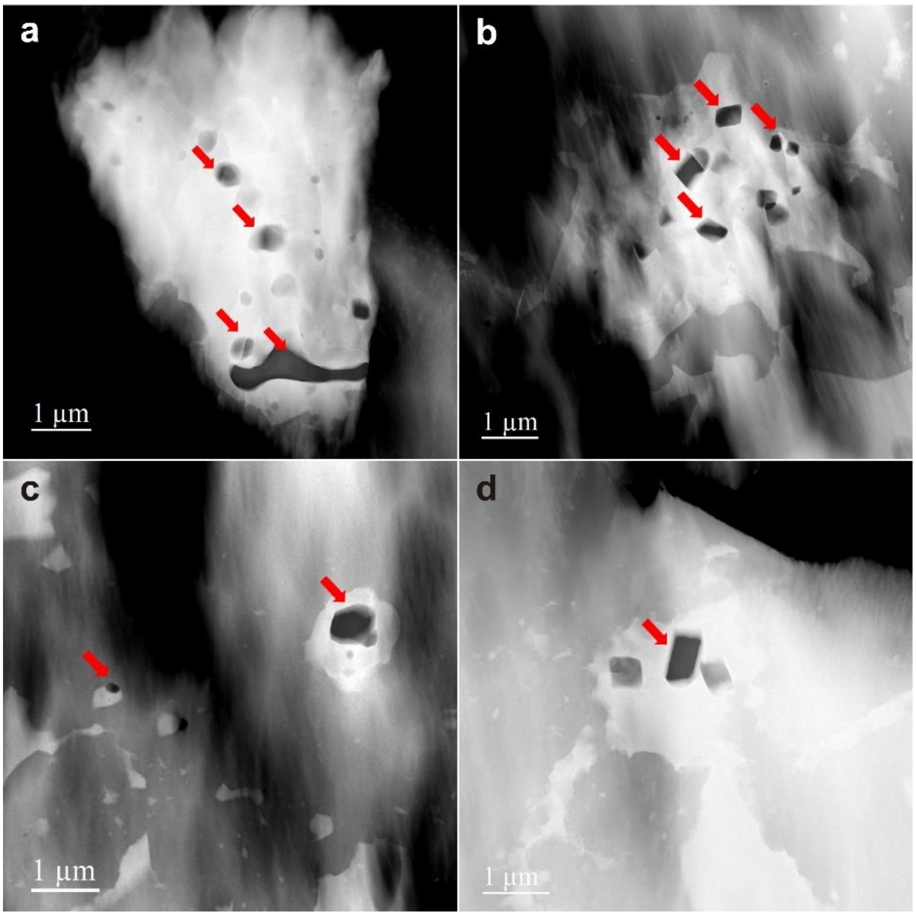

Supplementary Fig. 8 | Existence and distributions of graphene. a-d, High-angle annular dark field (HAADF)-Scanning transmission electron microscopy (STEM) images showing different areas containing graphene in 0.9Cu_2Se-0.1BPCCSO-0.035 wt% graphene composite. The red arrows marked the location of graphene.

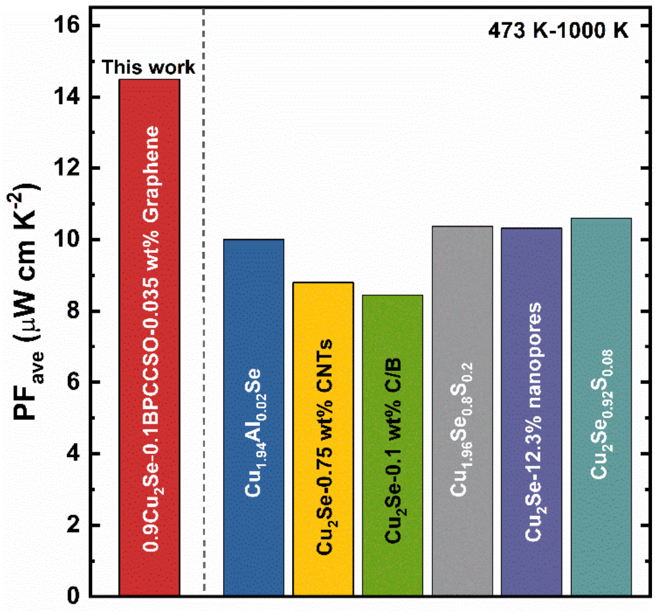

Supplementary Fig. 9 | Excellent electrical performance. Comparison on average power factor (PF_{ave} at 473 K to 1000 K) of 0.9Cu_2Se-0.1BPCCSO-0.035 wt% graphene composite in this work and other state-of-the-art Cu_2Se-based systems[3-8].

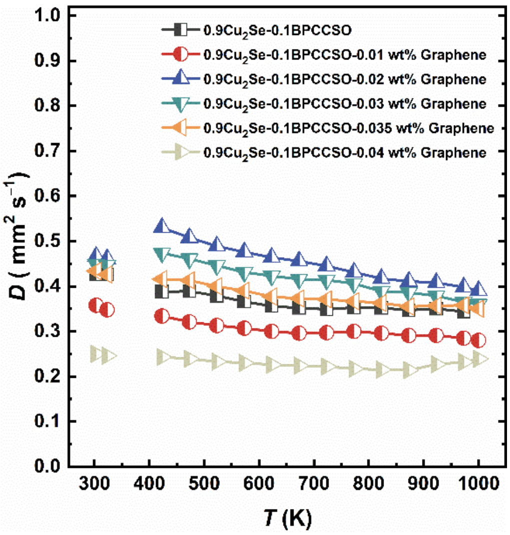

Supplementary Fig. 10 | Temperature-dependent thermal diffusivity (*D*) of 0.9Cu_2Se-0.1BPCCSO-x wt% graphene composites (x=0, 0.01, 0.02, 0.03, 0.035, 0.04). The thermal diffusivity values of all samples were measured by a laser flash method (LFA 457, Netzsch, Germany) from room temperature to 1000 K under a continuous argon flow.

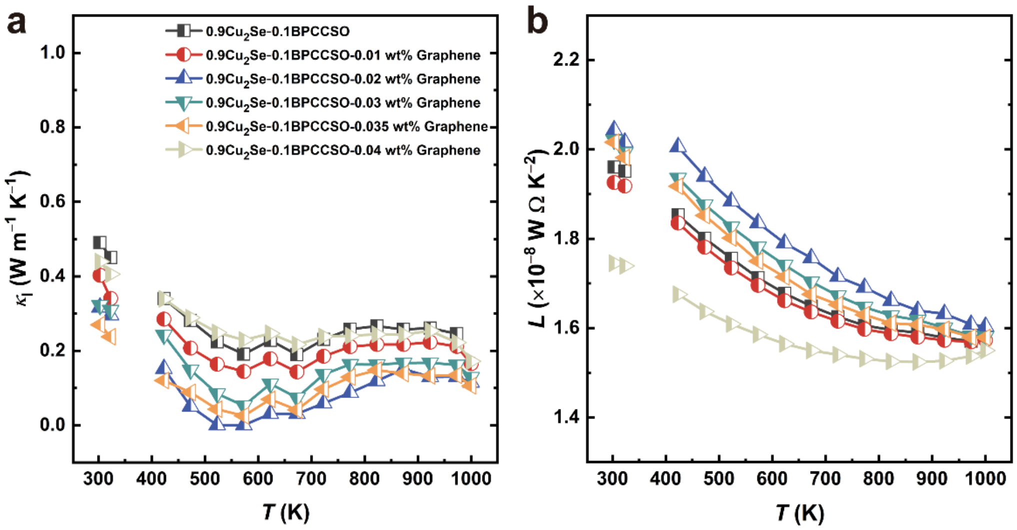

Supplementary Fig. 11 | Temperature-dependent thermal properties of 0.9Cu_2Se-0.1BPCCSO-x wt% graphene composites (x=0, 0.01, 0.02, 0.03, 0.035, 0.04). a, Lattice thermal conductivity (κ_l). **b,** Lorentz number (L).

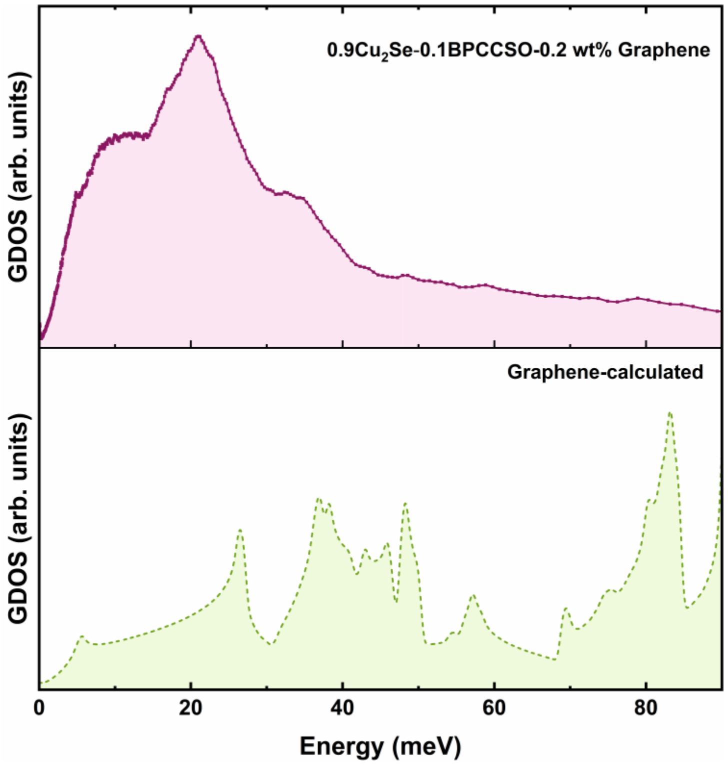

Supplementary Fig. 12 | Generalized phonon density of states (GDOS). The experimental GDOS of 0.9Cu_2Se-0.1BPCCSO-0.2 wt% graphene (300 K) and calculated GDOS of graphene.

Supplementary Tables 1-3

Supplementary Table 1 | Sample densities. The sample densities for 0.9Cu_2Se-0.1BPCCSO-x wt% graphene composites (x=0, 0.01, 0.02, 0.03, 0.035, 0.04), Cu_2Se-BPCCSO composites, BPCCSO and Cu_2Se. The relative density of each sample was over 95%. The declined density of 0.9Cu_2Se-0.1BPCCSO-x wt% graphene was consistent with the increasing graphene content.

Samples	ρ (g cm^{-3})
BPCCSO	8.446
Cu_2Se	6.690
0.7Cu_2Se-0.3BPCCSO	7.330
0.8Cu_2Se-0.2BPCCSO	7.137
0.9Cu_2Se-0.1BPCCSO	6.952
0.9Cu_2Se-0.1BPCCSO-0.01 wt% Graphene	6.949
0.9Cu_2Se-0.1BPCCSO-0.02 wt% Graphene	6.939
0.9Cu_2Se-0.1BPCCSO-0.03 wt% Graphene	6.926
0.9Cu_2Se-0.1BPCCSO-0.035 wt% Graphene	6.882
0.9Cu_2Se-0.1BPCCSO-0.04 wt% Graphene	6.834

Supplementary Table 2 | Hall measurement results. Room temperature Hall measurement results for $0.9Cu_2Se$-0.1BPCCSO-x wt% graphene composites (x=0, 0.01, 0.02, 0.03, 0.035, 0.04), BPCCSO and Cu_2Se, including Hall coefficient (R_H), carrier concentration (p) and Hall mobility (μ_H).

	R_H ($\times 10^{-2}$ cm^3 c^{-1})	p ($\times 10^{20}$ cm^{-3})	μ_H (cm^2 V^{-1} s^{-1})
BPCCSO	1.42	4.39	8.38
Cu_2Se	2.81	2.22	23.42
$0.9Cu_2Se$-0.1BPCCSO	2.23	2.80	25.24
$0.9Cu_2Se$-0.1BPCCSO-0.01 wt% Graphene	2.45	2.54	24.19
$0.9Cu_2Se$-0.1BPCCSO-0.02 wt% Graphene	2.57	2.43	39.63
$0.9Cu_2Se$-0.1BPCCSO-0.03 wt% Graphene	2.54	2.43	37.73
$0.9Cu_2Se$-0.1BPCCSO-0.035 wt% Graphene	2.58	2.42	38.12
$0.9Cu_2Se$-0.1BPCCSO-0.04 wt% Graphene	2.98	2.10	13.03

Supplementary Table 3 | Phonon transport properties. Sound velocities (longitudinal sound velocity (v_l), shear sound velocity (v_s) and average sound velocity (v_a)) of 0.9Cu_2Se-0.1BPCCSO-x wt% graphene composites (x=0, 0.01, 0.02, 0.03, 0.035, 0.04), BPCCSO and Cu_2Se. The elastic properties (Young's modulus (E), Poisson ratio (v_p), Grüneisen parameter (γ)) and mean free path (l_{ph}) were calculated based on sound velocity measurement.

	v_l (m s^{-1})	v_s (m s^{-1})	v_a (m s^{-1})	E (GPa)	ν_p	γ	l_{ph} (nm)
BPCCSO	3777	1947	2180	84.5	0.32	1.9	0.60
Cu_2Se	3243	1399	1581	36.3	0.39	2.5	0.40
0.9Cu_2Se-0.1BPCCSO	3150	1405	1585	37.8	0.38	2.4	0.34
0.9Cu_2Se-0.1BPCCSO-0.01 wt% Graphene	2846	1307	1473	32.4	0.37	2.3	0.30
0.9Cu_2Se-0.1BPCCSO-0.02 wt% Graphene	2854	1298	1463	32.0	0.37	2.3	0.24
0.9Cu_2Se-0.1BPCCSO-0.03 wt% Graphene	2854	1310	1476	32.5	0.37	2.3	0.24
0.9Cu_2Se-0.1BPCCSO-0.035 wt% Graphene	2831	1318	1484	32.6	0.36	2.2	0.20
0.9Cu_2Se-0.1BPCCSO-0.04 wt% Graphene	2968	1361	1534	34.6	0.37	2.3	0.32

Supplementary References

1. Snyder, G. J. et al. Weighted mobility. *Adv. Mater.* **32,** 2001537 (2020).

2. He, W. et al. High thermoelectric performance in low-cost $SnS_{0.91}Se_{0.09}$ crystals. *Science* **365,** 1418-1424 (2019).

3. Li, M. et al. Ultrahigh figure-of-merit of Cu_2Se incorporated with carbon coated boron nanoparticles. *InfoMat.* **1,** 108-115 (2019).

4. Nunna, R. et al. Ultrahigh thermoelectric performance in Cu_2Se-based hybrid materials with highly dispersed molecular CNTs. *Energy Environ. Sci.* **10,** 1928-1935 (2017).

5. Yang, D. et al. Blocking ion migration stabilizes the high thermoelectric performance in Cu_2Se composites. *Adv Mater.* 32, 2003730 (2020).

6. Zhao, K. et al. Enhanced thermoelectric performance through tuning bonding energy in $Cu_2Se_{1-x}S_x$ liquid-like materials. *Chem. Mater.* **29,** 6367-6377 (2017).

7. Mao, T. et al. Enhanced thermoelectric performance and service stability of Cu_2Se via tailoring chemical compositions at multiple atomic positions. *Adv. Funct. Mater.* **30,** 1908315 (2020).

8. Zhong, B. et al. High superionic conduction arising from aligned large lamellae and large figure of merit in bulk $Cu_{1.94}Al_{0.02}Se$. *Appl. Phys. Lett.* **105,** 123902 (2014).
